# Transcriptional profiles of circulating tumor cells reflect heterogeneity and treatment resistance in advanced prostate cancer

**DOI:** 10.1186/s13046-025-03367-x

**Published:** 2025-04-03

**Authors:** Lina Bergmann, Sarah Greimeier, Sabine Riethdorf, Tina Rohlfing, Moritz Kaune, Tobias Busenbender, Nadja Strewinsky, Sergey Dyshlovoy, Simon Joosse, Sven Peine, Klaus Pantel, Gunhild von Amsberg, Stefan Werner

**Affiliations:** 1 Institute of Tumor Biology, University Medical Center Hamburg-Eppendorf, Martinistrasse 52, 20246 Hamburg, Germany; 2 European Liquid Biopsy Society (ELBS), Hamburg, Germany; 3 Department of Hematology and Oncology, University Cancer Center Hamburg, University Medical Center Hamburg-Eppendorf, Martinistrasse 52, 20246 Hamburg, Germany; 4 Martini-Klinik, Prostate Cancer Center, University Medical Center Hamburg-Eppendorf, Martinistrasse 52, 20246 Hamburg, Germany; 5 Mildred Scheel Cancer Career Centre HaTriCS4, University Medical Centre Hamburg- Eppendorf, Martinistrasse 52, 20246 Hamburg, Germany; 6 Department of Transfusion Medicine, University Medical Center Hamburg-Eppendorf, Martinistrasse 52, 20246 Hamburg, Germany

## Abstract

**Purpose:**

New biomarkers for the detection and monitoring of aggressive variant prostate cancer (AVPC) including therapy-induced neuroendocrine prostate cancer (NEPC) are urgently needed, as measuring prostate-specific antigen (PSA) is not reliable in androgen-indifferent diseases. Molecular analysis of circulating tumor cells (CTC) enables repeated analysis for monitoring and allows to capture the heterogeneity of the disease.

**Experimental design:**

102 blood samples from 76 metastatic prostate cancer (mPC) patients, including 37 samples from histologically proven NEPC, were collected and CTCs were enriched using label-dependent and label-independent methods. Relevant transcripts were selected for CTC profiling using semi-quantitative RT-PCR analysis and validated in published datasets and cell lines. Transcriptional profiles in patient samples were analyzed using supervised and unsupervised methods.

**Results:**

CTC counts were increased in AVPC and NEPC as compared to metastatic hormone-sensitive prostate cancer (mHSPC). Gene expression profiles of CTCs showed a high degree of inter-patient heterogeneity, but NEPC-specific transcripts were significantly increased in patients with proven NEPC, while adenocarcinoma markers were decreased. Unsupervised analysis identified four distinct clusters of CTC^low^, AR^high^, amphicrine and pure NEPC gene expression profiles that reflected the clinical groups. Based on the transcript panel, NEPC could be distinguished from mHSPC or AVPC patients with a specificity of 95.5% and 88.2%, respectively.

**Conclusion:**

Molecular subtypes of mPC can be distinguished by transcriptional profiling of CTCs. In the future, our convenient PCR-based analysis may complement the monitoring of advanced PCa patients and allow timely detection of resistance to androgen receptor pathway inhibitors.

**Supplementary Information:**

The online version contains supplementary material available at 10.1186/s13046-025-03367-x.

## Introduction

Treatment and monitoring of prostate cancer (PC) patients is primarily based on the androgen receptor (AR) signaling pathway. The gene product of the AR target gene *KLK3* encoding the prostate-specific antigen (PSA) protein is commonly used for PC screening and disease monitoring [1]. Androgen receptor pathway inhibitors (ARPI) such as the anti-androgens apalutamide, enzalutamide and darolutamide block AR translocation and binding and are approved for the treatment of advanced stages of PC [2]. However, increased therapeutic pressure on the AR signaling transduction pathway is associated with an increased frequency of therapy-induced AR-independent PC including aggressive variant prostate cancer (AVPC) [3, 4]. The presentation of AVPC is highly variable. Aparicio and colleagues defined seven criteria to identify these patients in the clinic, including visceral metastasis, short interval to androgen-independent progression or low PSA at progression [5]. The emergence of therapy-induced NEPC as a phenotype resistant to ARPI is a consequence of highly effective and prolonged AR inhibition and is caused by epigenetic and transcriptional reprogramming [6]. Using transcriptional profiling of a large collection of mPC tumor lesions and preclinical model systems, other investigators have found defined molecular subtypes of treatment resistance in metastatic tumor lesions, including tumors that retain strong activation of AR signaling, tumors with gene expression profiles of NEPC, an amphicrine subtype positive for both AR and NEPC markers, and double-negative PC (DNPC), which lack expression of either AR- or NEPC-specific transcripts. In this context, detection of NEPC features and absence of AR signaling may indicate androgen-independent disease, which is resistant to ARPI [7].

In everyday clinical practice, the diagnosis of androgen-independent disease progression is often made too late, as monitoring is frequently only PSA-based. Many patients are not diagnosed until a general deterioration occurs and are then often no longer fully accessible to effective treatment options [8]. Additional biomarkers are urgently needed to improve the diagnosis of emerging AVPC and therapy-induced NEPC, to avoid ineffective treatments and to guide more appropriate, usually chemotherapy-based, treatment strategies [5]. CTC enumeration in peripheral blood has been shown to be particularly useful in monitoring mPC patients, including therapy-induced NEPC [9–11]. Previously, customizable liquid biopsy assays for gene expression analysis of CTCs have been successfully applied, while single transcripts are promising CTC-based biomarkers for detection of therapy-induced NEPC [12–14].

In this study, we determined CTC counts in blood samples of mPC patients and developed a semi-quantitative PCR-based method to detect AR activation and NE traits in enriched CTCs. Gene expression analysis revealed four discrete clusters of patient samples that reflected the histology of the corresponding tumor lesions. Using a random forest classifier, we could robustly identify samples from NEPC patients with high sensitivity and specificity. We hypothesize that by using the developed comprehensive multi-marker panel in clinical practice, it can help to identify patients with emerging or manifest therapy-induced NEPC and thus can help to guide treatment decisions.

## Materials and methods

A total of 103 blood samples were prospectively collected from patients with mPC at the University Medical Center Hamburg-Eppendorf between November 2020 and July 2023 in accordance with the Declaration of Helsinki and approved by the Ethics Committee of the City of Hamburg (Hamburger Ärztekammer, PV5392). Patient characteristics are given in Supplementary table S1. All patients gave informed consent. They were male, aged between 46 and 88 years, and were receiving different therapies. No blinding, randomization or exclusion criteria were used and there was no attrition from the study. Because this was a pilot study, a formal power calculation was omitted due to unknown effect size.

7.5 ml of peripheral blood was collected in either EDTA or CellSafe tubes and processed within 3 h. Blood from age-matched male donors was used as a negative control. CTC enumeration was performed using the Cellsearch system and gene expression was performed on the bulk CTC fraction enriched with the AdnaTest Prostate Cancer Select Kit. Patients were grouped as AVPC according to the criteria of Aparicio et al. [5]. From the AVPC patient cohort, patients with histological confirmed NEPC were evaluated separately. In addition, patients without adenocarcinoma or neuroendocrine marker expression in histological staining (“double negative) were analyzed alone [15]. Assays were performed blinded to groups. CTC counts and differential gene expression between groups were analyzed using Kruskal-Wallis test and Wilcoxon rank sum test with Dunn’s correction for multiple testing for pairwise comparisons. Chi-squared test and Fisher’s exact test were used to assess differences between categorical variables, such as the positivity of a particular marker per group, while clustering based on gene expression profiles was performed using the ward.D method with Euclidean as the distance measure. A detailed description of all materials and methods used in this study can be found in the supplementary information.

## Results

### Detection of CTCs in mPC patients

**Fig. 1:**
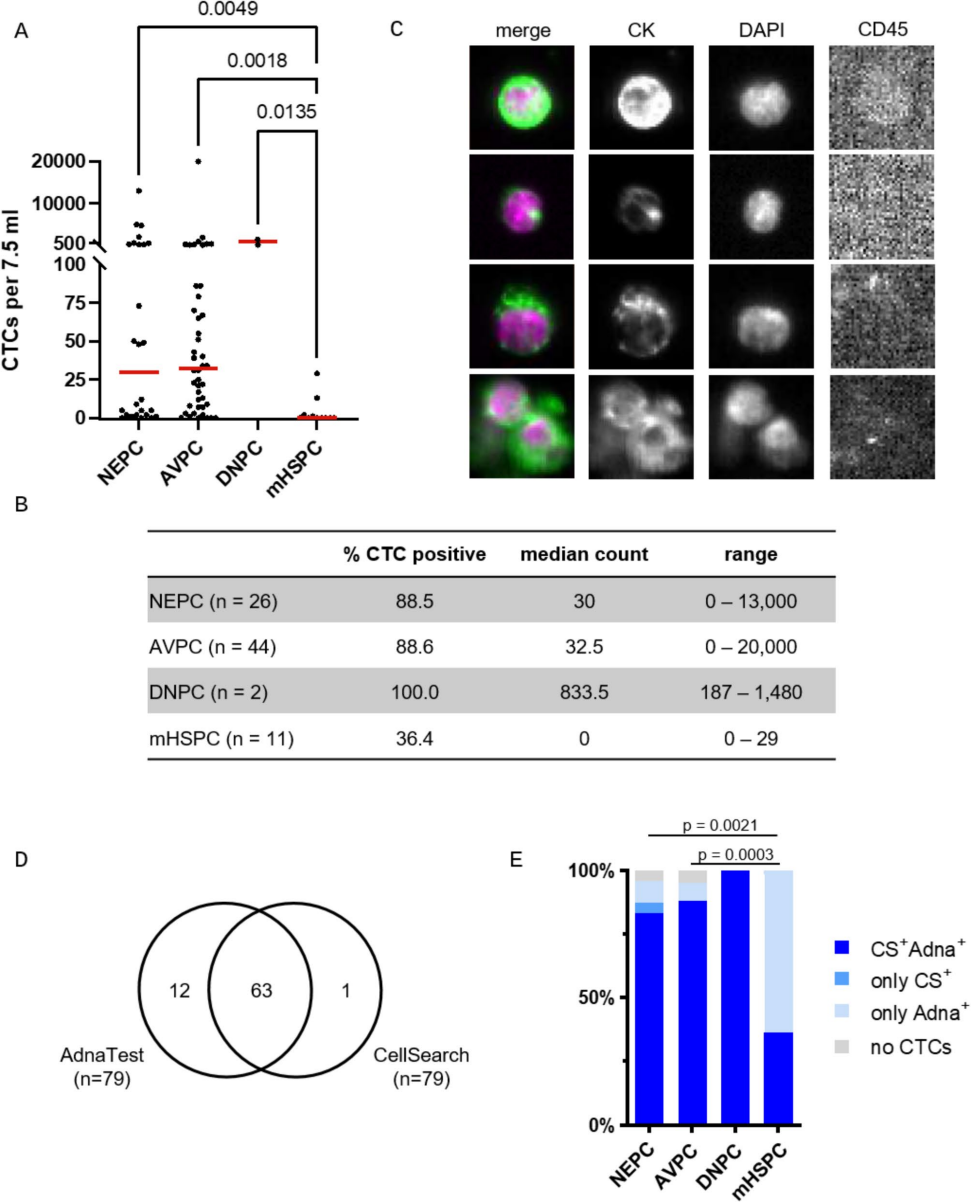
CTC detection in advanced PC patients. CTCs were analysed from blood samples of PC patients with AVPC, NEPC, DNPC or mHSPC with CellSearch or AdnaTest; CTCs were defined by positive CK staining and absent CD45 staining in CellSearch and by detection of epithelial or adenocarcinoma markers in AdnaTest; **A**: CTC count of individual samples per group, red line indicates the group median; **B**: representative images of CTCs detected by CellSearch; **C**: CTC positivity, median CTC count and range determined per group by CellSearch; **D**: Venn diagram illustrating the number of CTC positive samples in parallel CellSearch and AdnaTest analysis; **E**: comparison of the percentage of CTC positive samples between the two enrichment methods and the four patient groups, CS-Cellsearch

We first enumerated CTCs using the FDA-cleared CellSearch analysis. 83 blood samples were available for this analysis (44 AVPC, 26 NEPC, 11 mHSPC, 2 DNPC). In the NEPC group, 88.5% of samples were positive for CTCs with a median count of 30 CTCs per 7.5 mL and a range of 0–13,000 CTCs (Fig. 1A-B). This was significantly higher than in the mHSPC group with 36.4% positive samples and a median CTC count of 0 CTCs per 7.5 mL (*p* = 0.0049). Similarly, the AVPC group showed a significantly increased CTC positivity of 88.6% with a median count of 32.5 CTCs per 7.5 mL and a range of 0–20,000 CTCs (*p* = 0.0018). There was no difference in CTC counts between NEPC and AVPC samples (*p* > 0.999). Both DNPC patients were CTC positive and had high counts of 187 and 1,480 CTCs per 7.5 ml. CTCs detected by CellSearch showed variable morphology (Fig. 1C). Some CTCs were characterized by a small-cell-like morphology with peri-nuclear, dot-like cytokeratin staining; however, the detection of these small-cell-like CTCs was not significantly different between the groups (Supplementary Figure S1).

The high CTC counts observed in mPC patients facilitate subsequent molecular analysis, but gene expression analysis is inapplicable after CellSearch analysis, which requires sample fixation. Therefore, we compared the CellSearch results to the AdnaTest Prostate Cancer, another immunomagnetic enrichment procedure that allows gene expression analysis [16]. Like CellSearch, the AdnaTest analysis is primarily based on EPCAM expression of CTCs, but also uses EGFR and HER2 for CTC enrichment [17]. Since enumeration of CTCs is not possible in AdnaTest, we compared the CTC positivity in samples processed by both methods in 79 parallel samples. CTC positivity in AdnaTest was based on the detection of at least one or combined expression of *EPCAM*,* KRT19*,* AR*,* KLK3*,* FOLH1*,* NKX3-1* and *HOXB13* genes. As these transcripts are not expressed in leukocytes [18, 19], their detection indicates the presence of CTCs in blood samples. 95% of the samples were positive in AdnaTest compared to 81% in CellSearch (*p* = 0.014, Fig. 1D). 13 samples were not concordant between both methods: one sample was negative in AdnaTest but not in CellSearch, while twelve samples were CTC negative in CellSearch and positive in AdnaTest. Differences in CTC positivity rates may be due to statistical variability in true CTC counts between two blood samples but may also be due to the two additional antigens used in the AdnaTest or non-specific detection of transcripts.

When comparing the four patient groups of mHSPC, AVPC, NEPC and DNPC, the concordance between AdnaTest and CellSearch was higher in the NEPC and AVPC groups, both of which have high CTC counts compared to the mHSPC group (*p* = 0.0021, *p* = 0.0003, Fig. 1E). While more than 85% of the CTC-positive samples in the NEPC and AVPC groups were CTC positive in both methods, only 36% of the CTC-positive samples in the mHSPC group were CTC-positive with both methods. The remaining 64% of samples were only CTC positive with the AdnaTest, indicating that both enrichment methods were robust in detecting CTCs, but the AdnaTest was more sensitive at lower cell numbers. In conclusion, using label-dependent enrichment we detected high numbers of CTCs in blood samples of AVPC and NEPC patients, facilitating subsequent molecular analysis.

### Development of a marker panel for the detection of NEPC

**Fig. 2:**
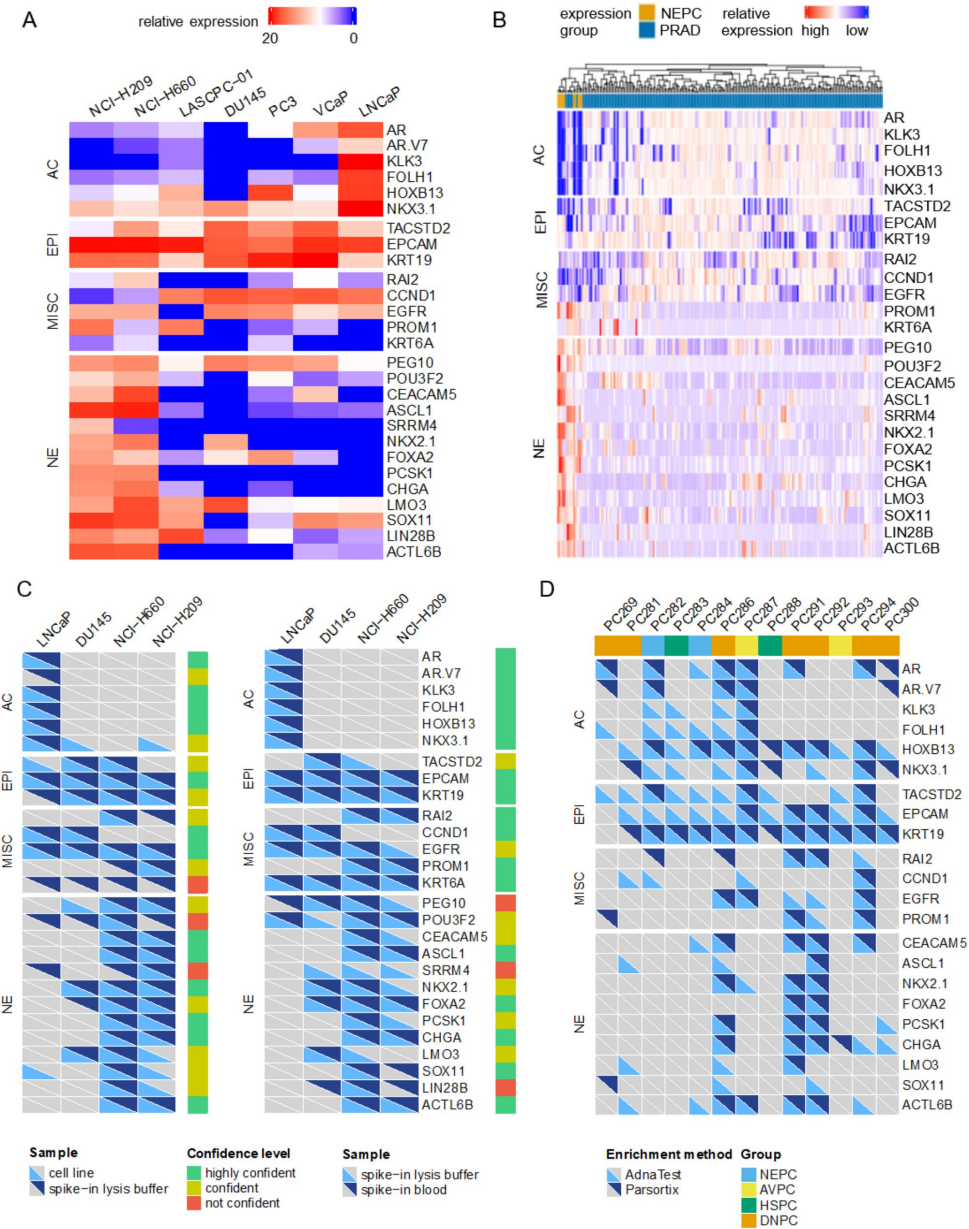
Validation of a marker panel for NEPC detection. **A**: normalized gene expression as reversed ΔC_q_ of candidate markers in PC and NE cell lines; **B**: hierarchical clustering of published PC tissue data from the Metastatic Prostate Adenocarcinoma data set [3] based on the selected marker panel; **C**: comparison of marker detection between pure cell lines and spike-in controls, 25 single cells of the indicated cell lines were spiked in AdnaTest lysis buffer or healthy donor blood and marker expression was detected by PCR, light blue– marker detected in cell line (left) or lysis buffer spike in (right), dark blue– marker detected in lysis buffer spike-in (left) or blood spike-in (right); **D**: Validation of marker set in parallel samples of label-dependent and label independent CTC enrichment, detection of a marker is indicated in light blue for the AdnaTest and dark blue for Parsortix; AC– adenocarcinoma, EPI– epithelial, MISC– miscellaneous, NE– neuroendocrine

Because CTC counts and morphology analysis were not sufficient to identify NEPC samples, we proceeded with gene expression analysis in enriched CTCs. The marker panel comprised previously published epithelial and adenocarcinoma markers [17]. Additionally, we performed literature research on PubMed (NCBI) with the terms “transdifferentiation”, “neuroendocrine prostate cancer” and “small cell prostate cancer” and filtered for research papers to identify molecular drivers and markers of NEPC, the complete results have been published previously [20]. To analyze only tumor-specific markers, transcripts that are expressed in leukocytes were excluded from the outset [18, 19]. In total we selected 13 NE markers, and 4 markers associated with stemness and therapy resistance for further validation that are not expressed in PBMCs (Supplementary Table S2). We examined the expression of the selected genes in six PC and one small-cell lung cancer cell line as additional reference for NE differentiation (Fig. 2A). Both NE cell lines, NCI-H660 and NCI-H209, showed similar expression patterns with upregulation of NE markers and downregulation of adenocarcinoma (AC) markers. The only hormone-sensitive cell line, LNCaP, was clearly distinguished by its high expression of AR signaling pathway genes and the absence of NE markers. DU-145 PC cells showed a mixed phenotype with downregulation of AC markers and only focal expression of NE markers. Next, we validated the marker panel in two published transcriptome data sets derived from CRPC tissue with annotation of NEPC status [9]. Hierarchical clustering of the patient samples based on the marker panel revealed precise clustering of the NEPC patients in the Metastatic Prostate Adenocarcinoma dataset [3] (Fig. 2B) and a distinct clustering in the NEPC data set [21] (Supplementary Figure S2).

We proceeded with the technical validation of the CTC enrichment and gene expression analysis workflow. A pre-amplification step was included to detect all transcripts in the small amounts of RNA derived from enriched CTCs. Comparison of the marker detection in the cell lines and the detection after pre-amplification RNA isolated from 25 cells revealed a high concordance (Fig. 2C). The markers *KRT6A*, *POU3F2* and *SRRM4* had to be excluded due to non-specific signals in the low input controls. Next, 25 cells were spiked into healthy donor blood, enriched, and the marker expression was compared to the unenriched control (Fig. 2C). While most markers were detected with high confidence, *PEG10* and *LIN28B*, had to be excluded from the panel as they showed a non-specific signal in the enriched samples. Thus, 22 out of 27 markers were used for subsequent analysis in human CTC samples.

As the use of EPCAM as a positive marker for CTC enrichment in the context of lineage plasticity is under debate [22], we further compared a label-independent CTC enrichment method with a label-dependent size-based method by Parasortix. 13 patient samples (7 NEPC, 2 AVPC, 2 mHSPC, 2 DNPC) were available for parallel analysis. After enrichment, samples underwent an identical workflow of RNA isolation, cDNA synthesis and PCR-based detection. Regarding the epithelial markers, 92% (12/13) of the samples were positive for at least one marker in both enrichment methods, whereas one sample was positive for one epithelial marker only in AdnaTest (Fig. 2D). The concordance of the individual marker-patient combinations was mixed, as 38.5% of the measurements were only positive in AdnaTest enriched samples and 5.1% only in Parsortix-enriched samples. AdnaTest may be more sensitive to enrich epithelial cells from the blood of the selected patients, while Parsortix might enrich dedifferentiated cells or lose small CTCs. Similar results were observed for the AC markers, which were also used to define samples as CTC positive. 77% (10/13) of the samples were positive for at least one AC marker in both tests, while two samples were positive for AC markers on the AdnaTest and one sample was positive only in the Parsortix.

38% (5/13) of the samples were positive for at least one NE marker with both enrichment methods. 23% (3/13) were only positive for NE markers after label-dependent enrichment, while 15% (2/13) were only detected in size-based enriched cells. Four of the five samples that were positive by both methods had more NE markers detected in label-dependent enriched CTCs. In all samples that were positive only after label-dependent enrichment, more than one NE marker was detected. In contrast, only a single marker was detected in those samples positive in Parsortix but not AdnaTest. 56.8% of detected markers were overlapping with both methods, 37.8% were only found after label-dependent enrichment and 5.4% only after size-based enrichment. As NE markers were more frequently detected after label-dependent enrichment (*p* = 0.0013), we conclude that this method is appropriate and superior to size-dependent enrichment for subsequent analysis of a larger patient cohort to detect patients with NEPC.

### Gene expression analysis of enriched CTCs.

**Fig. 3:**
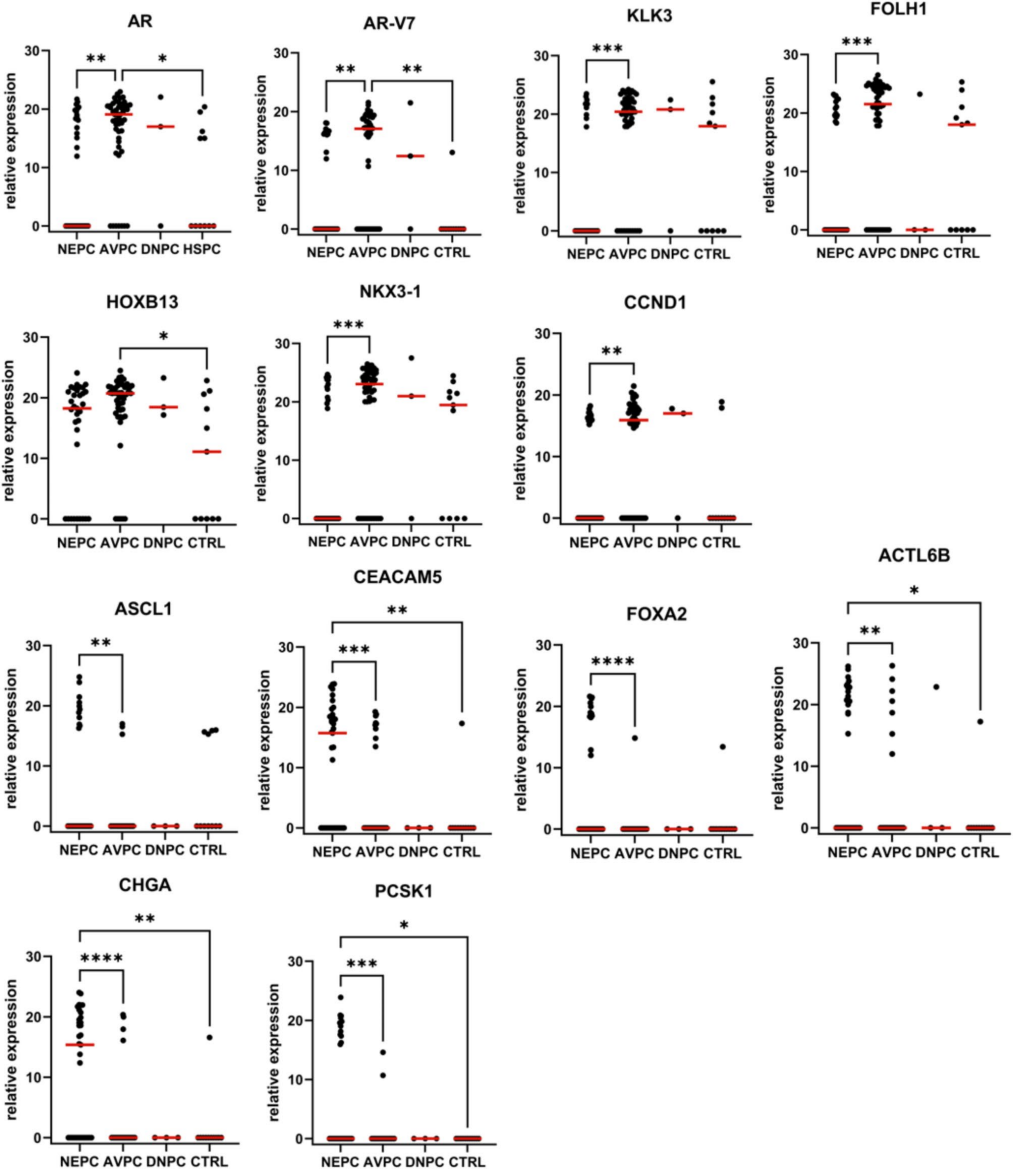
Expression of indicated genes in enriched CTC fractions from advanced PC patients. CTCs were enriched from blood samples of PC patients using the AdnaTest Prostate Cancer Select and a panel of AC and NE markers was detected following multiplex pre-amplification of isolated RNA; samples were grouped according to clinical and histopathological parameters into neuroendocrine PC (NEPC), aggressive variant PC (AVPC), double-negative PC (DNPC) and metastatic hormone-sensitive PC (mHSPC); normalized gene expression is shown as reversed ΔC_q_ and the Kruskal Wallis Test together with Wilcoxon rank sum test and Dunn’s correction for multiple testing for pair-wise comparison, **A**: adenocarcinoma markers, **B**: neuroendocrine markers

In total of 99 samples gene expression of the selected marker panel was determined in enriched CTCs and patients were stratified as AVPC, NEPC, DNPC, and mHSPC as above (Supplementary Table S1). The expression of *EPCAM*, *KRT19*, and *TACSTD2* showed no differences between the groups (Supplementary Figure S3). Genes with significantly different expression levels between the groups are shown in Fig. 3. *AR*, was detected in most of the CTC samples and the expression in the AVPC group was significantly higher than in the NEPC and the mHSPC groups (*p* = 0.0012; *p* = 0.0265). Although expressed in fewer samples overall, the *AR-V7* splice variant showed a similar pattern with increased expression in AVPC compared to NEPC and mHSPC samples (*p* = 0.0017; *p* = 0.0015), which may be due to lower CTC counts in this group. Gene expression of the AR targets *KLK3*, *FOLH1*, and *NKX3-1* was detected in all groups, while the expression was significantly reduced in NEPC compared to AVPC (*p* = 0.002; *p* = 0.0004; *p* = 0.0002). The *HOXB13* gene was upregulated in AVPC compared to mHSPC (*p* = 0.0455). No significant difference was found between AVPC and NEPC (*p* = 0.1489). Like the AC markers, the cell cycle regulator *CCND1* was downregulated in NEPC compared to AVPC (*p* = 0.0084). A strong positive correlation was observed between the expression levels of the individual AC markers, *CCND1* and epithelial marker expression (Supplementary Figure S6).

Significant differences between the groups were found for six out of nine NE markers. *CHGA* was one of the most abundantly expressed NE markers and its expression was increased in NEPC samples compared to mHSPC and AVPC samples (*p* = 0.0065; *p* < 0.0001). Similarly, *CEACAM5* was higher expressed in NEPC samples compared to mHSPC and AVPC (*p* = 0.0081; *p* = 0.0003). *ACTL6B* was another marker with increased expression in NEPC compared to mHSPC and AVPC (*p* = 0.04; *p* = 0.0019). *ASCL1* was increased in NEPC compared to AVPC, although only expressed in a minority of samples (*p* = 0.0072). In addition, *PCSK1* and *FOXA2* were induced in NEPC compared to AVPC (*p* = 0.0003; *p* < 0.0001). The detection of *LMO3*, *SOX11*, and *NKX2-1* transcripts did not show significant differences between the groups. The expression of the individual NE markers was mostly correlated with each other, while only *ASCL1*, *PCSK1*, and *ACTL6B* expression was anti-correlated with one or more of the AC markers *KLK3*, *FOLH1*, and *CCND1* (Supplementary Figure S4). In summary, the comparison of individual markers highlights the downregulation of AC markers and the induction of NE markers in CTCs from NEPC patients.

**Fig. 4:**
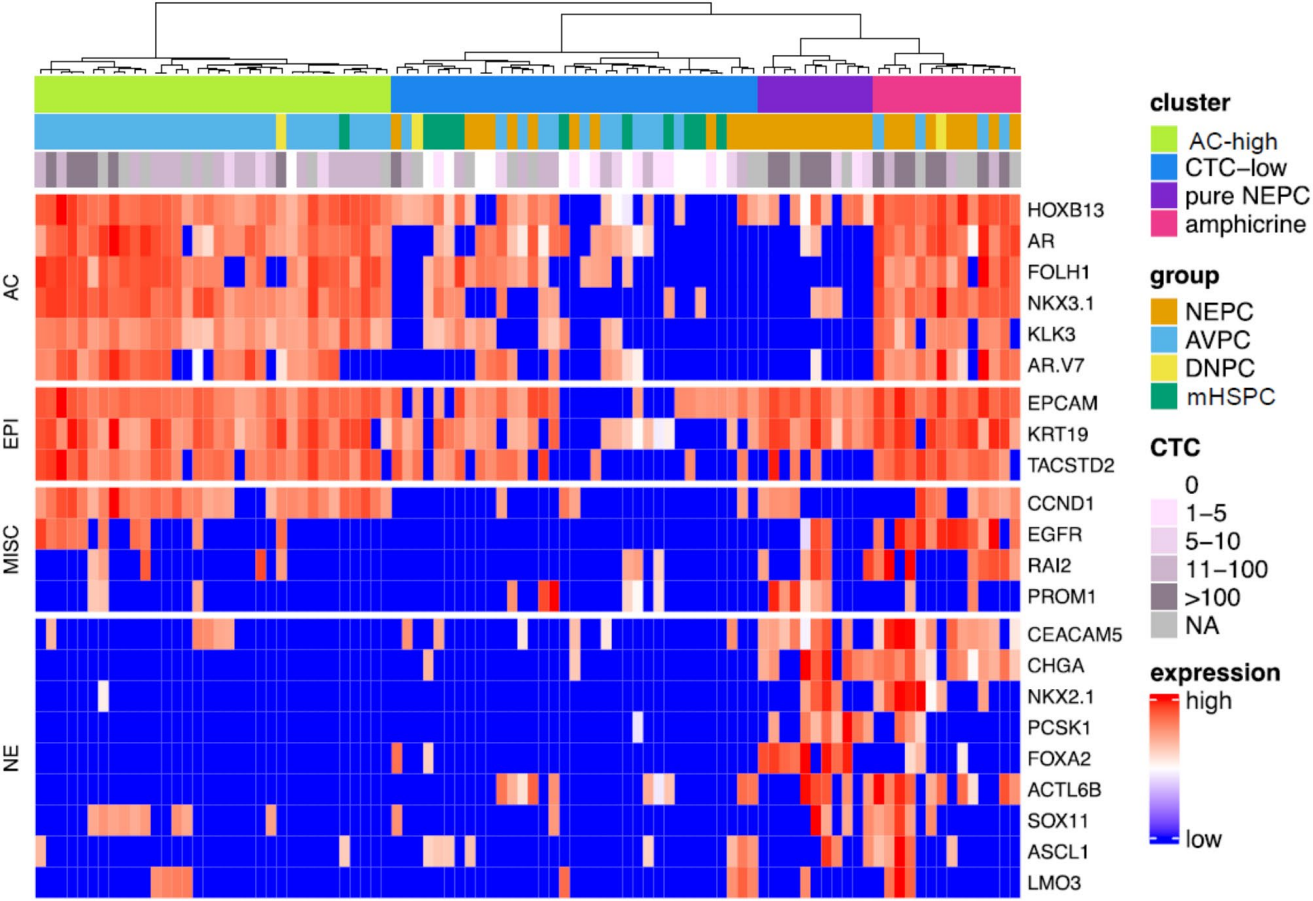
Hierarchical clustering of patient samples based on CTC gene expression profiles. Normalized C_q_ values were scaled and subjected to hierarchical clustering based on the relative expression of all markers in the panel; based on the dendrogram, samples were split into four clusters as indicated on the right; the group and the CellSearch CTC count for each sample are shown on top of the histogram

To identify treatment-relevant subgroups, we performed hierarchical clustering based on the expression of the marker panel; 94 samples with complete expression profiles were included and the analysis identified four distinct clusters (Fig. 4). The first cluster included 34 samples, and the expression profile was dominated by a high expression of AC and epithelial markers. With a few exceptions, NE gene expression was almost absent in samples from this cluster. This cluster was termed the AR^high^ cluster, with samples predominantly showing clinical features of AVPC. None of these samples are part of the NEPC group and the samples in this cluster had high CTC counts with a median of 40. The second cluster included 35 samples with mixed expression of PRAD markers and reduced to absent expression of epithelial markers. Also, considering the low median CTC count of 1.5 CTCs per 7.5 ml of blood, this cluster was designated the CTC^low^ cluster. Consistent with this, NE markers were also negative in this cluster, except for individual samples with expression of few NE markers. Samples in the CTC^low^ cluster were from all clinical groups, suggesting that this cluster does not represent a clinical subtype but rather an overall CTC^low^ phenotype, which is also reflected by low expression of *EPCAM*, *KRT19* and *TACSTD2* as a putative surrogate for CTC count. However, individual samples from this cluster showed abundant CTC counts in the absence of AC and NE gene expression, likely indicating the emergence of DNPC. The third cluster consisted of eleven samples and was characterized by the absence of AC markers except for *HOXB13*. Epithelial markers *EPCAM* and *KRT19* were highly expressed, while *TACSTD2* was downregulated compared to the AR^high^ and the amphicrine cluster (*p* = 0.006; *p* = 0.0044). NE markers were highly expressed in this cluster, with all samples being positive for multiple markers. Expression of *FOXA2* and *PCSK1* were specific for this cluster showing increased expression compared to the other three clusters (*p* < 0.0001; *p* < 0.0001). This cluster was therefore considered the pure NEPC cluster and included only samples with histological evidence of NEPC. The stemness marker *PROM1* was enriched in this cluster compared to the AR^high^ and the amphicrine cluster (*p* = 0.0028; *p* = 0.0401). Samples in the fourth cluster shared a double-positive expression profile with intense expression of AC markers and detection of multiple NE markers, predominantly *CEACAM5*, *CHGA* and *ACTL6B*, and was therefore named amphicrine. It was also characterized by increased *EGFR* expression compared to all other clusters (*p* < 0.0001). Patients assigned to the amphicrine cluster were classified either as NEPC or AVPC based on their clinical parameters, highlighting the difficulty of stratifying patients with mixed tumors in the clinic. Thus, hierarchical clustering identified four clusters with distinct patterns of gene expression that resembled the clinical groups.

### NEPC can be predicted based on CTC gene expression

**Fig. 5:**
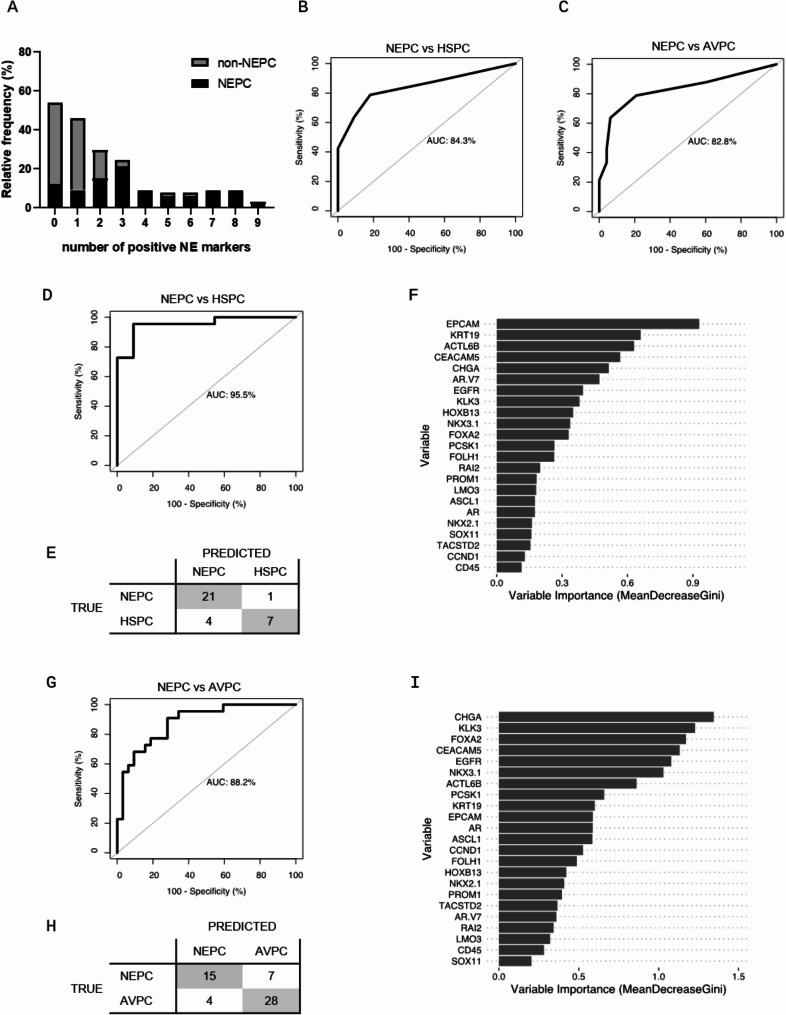
Classification of NEPC samples based on CTC gene expression profiles. **A**: normalized gene expression data were simplified to binary representation of marker positivity based on thresholds set by means of healthy donor samples, the frequency distribution of the number of positive NE markers in CTC positive samples is shown for NEPC compared to non-NEPC (AVPC, mHSPC, DNPC); **B**: ROC analysis of the count of positive NE markers as a marker to distinguish between NEPC and mHSPC, **C**: ROC analysis of the count of positive NE markers as a marker to distinguish between NEPC and AVPC; **D**: ROC analysis of the random forest classifier trained on normalized gene expression data of NEPC and mHSPC samples from individual patients positive for CTCs; **E**: results of the leave-one-out cross-validation included in the classifier training; **F**: variable importance of the individual markers included in the classifier; **G**: ROC analysis of the random forest classifier trained on normalized gene expression data of NEPC and AVPC samples from individual patients positive for CTCs; **H**: results of the leave-one-out cross-validation included in the classifier training; **I**: variable importance of the individual markers included in the classifier

We next aimed to predict the NEPC samples based on the expression of the markers in our panel. First, we evaluated the number of positive NE markers as a simple measure to identify NEPC patients. The non-NEPC samples, including AVPC, DNPC and mHSPC, expressed a significantly lower number of NE markers with a median of 1 positive marker compared to 3 positive markers in the NEPC group (*p* < 0.0001, Fig. 5A). The number of positive NE markers was used as a classifier in a ROC analysis to discriminate between NEPC from AVPC and mHSPC, respectively. NEPC and mHSPC could be discriminated with an AUC of 84.3% and a specificity 81.82% and sensitivity of 78.79% at a threshold of two or more positive NE markers (Fig. 5B). Similarly, the number of positive markers was sufficient to discriminate between NEPC and AVPC with an AUC of 82.8% (Fig. 5C).

To test whether the sample group could be predicted with even higher accuracy, a random forest model with leave-one-out cross-validation was trained to include all markers in the panel. To avoid overfitting, only one sample per patient was included if multiple samples were collected. Only samples that were CTC-positive based on the AdnaTest results were included in the analysis to avoid training of the classifier to identify CTC positive samples in general. First, the model was trained to discriminate between NEPC and mHSPC patients based on a subset of 22 NEPC and 11 mHSPC patients. This resulted in a classifier that could predict the group with an AUC of 95.5% and a sensitivity of 90.9% at a specificity of 95.5% (Fig. 5D). Cross-validation showed an error rate of 15.15%. Misclassified samples were predominantly in the mHSPC group and had borderline probabilities for both groups (Fig. 5E). Two mHSPC samples were positive for *ASCL1* and might have therefore been predicted to be NEPC. As histopathological analysis did not show traits of NEPC, stricter thresholds for marker detection might be required. The two other mHSPC samples were reported to have low or partial PSA staining on histology and could be followed up more closely. Another misclassified mHSPC sample was positive for only one epithelial marker, and it remains questionable whether this sample was positive for CTCs at all. The analysis of variable importance revealed that the epithelial markers *EPCAM* and *KRT19* were the most important features for group assignment (Fig. 5F). This emphasizes the maintained epithelial differentiation in the NEPC group and might be due to the high CTC counts observed in this group compared to mHSPC patients (Fig. 1A). The NE markers *ACTL6B*, *CEACAM5* and *CHGA* were also among the highly ranked variables for NEPC predictions. However, the addition of the CTC count did not improve the performance of the random forest model (Supplementary figure S5).

After successful discrimination between NEPC and mHSPC patients, we trained another model to discriminate between NEPC and AVPC samples, which are more difficult to detect clinically. 22 NEPC and 32 AVPC samples were included in the analysis. The model was able to discriminate between the two groups with an AUC of 88.2%, 23.53% samples were misclassified with a trend to incorrectly identify NEPC samples as AVPC and not vice versa (Fig. 5G and H). Closer examination of the misclassified patients revealed that these cases were mostly assigned to the amphicrine cluster with double-positive marker expression. Other misclassified samples from both the AVPC and the NEPC groups belonged to the CTC^low^ cluster, suggesting that marker detection in those samples may have been hampered by the low CTC counts. The most important variables in the model were *CHGA* and *KLK3* (Fig. 5I), emphasizing the need for PRAD as well as NE markers for prediction and simultaneously explaining the difficulties in the classification of double-positive samples. As our analysis is based on gene expression analysis of bulk enriched CTC, it remains unknown whether the amphicrine gene expression signature results from pure amphicrine tumor lesions or whether this signature is the result of admixed PRAD and NEPC CTCs. In conclusion, although the origin of the amphicrine gene expression in CTC remains unclear, we were able to robustly discriminate between NEPC and mHSPC or AVPC subtypes based on the detection of AC and NE markers in enriched CTCs.

### Longitudinal CTC analysis in a therapy-induced NEPC case

**Fig. 6:**
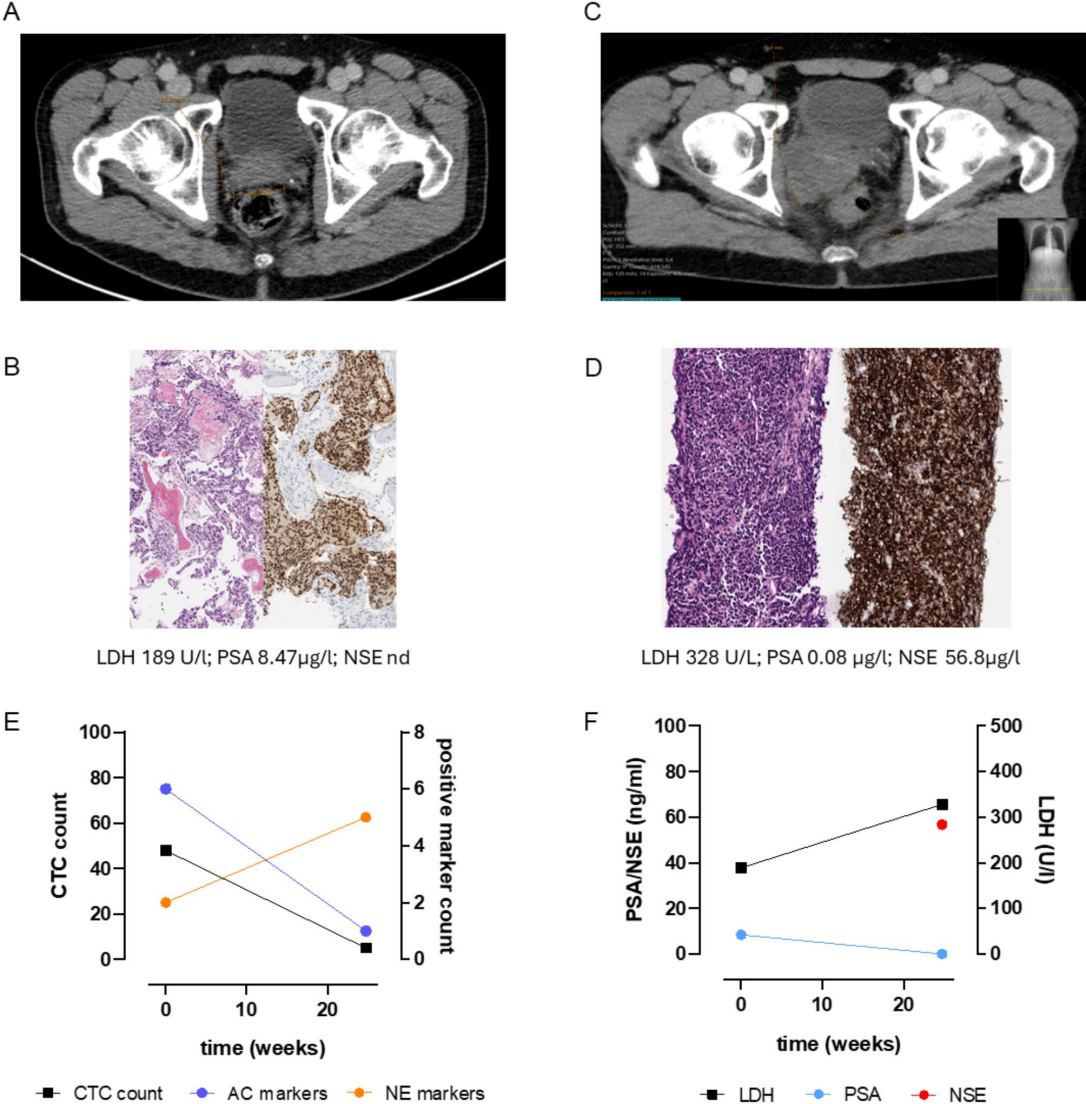
Case study of a patient with longitudinal CTC analysis. **A**: Computer tomography of the pelvis at diagnosis; **B**: computer tomography of the pelvis with local, PSA-negative progression of the primary; **C**: Histological confirmation of an adenocarcinoma of the prostate from a myelon-compressing bone metastasis (left HE staining, right NKX3.1 immunohistochemistry) and serum markers at the time of biopsy; **D**: Histological confirmation of a neuroendocrine (NE) transdifferentiation from the primarius (left HE staining, right synaptophysin immunohistochemistry) and serum markers at the time of biopsy; **E**: CTC count determined by CellSearch and the numbers of positive adenocarcinoma (AC) and NE markers after AdnaTest-based gene expression analysis at the first and the second timepoint of blood collection; **F**: serum markers lactate dehydrogenase (LDH), prostate specific antigen (PSA) and neuron-specific enolase (NSE) at the first and second timepoint of blood collection

Although longitudinal analysis was not a predefined study objective, repeated blood samples were available for some patients. In fact, one mHSPC transdifferentiated to NEPC during the study period. At initial diagnosis, the patient presented neurological symptoms due to spinal cord compression of a primary osseous metastatic PCa. Decompression surgery revealed an adenocarcinoma originating from the prostate. Representative images of the bone metastasis biopsy are given in Fig. 6A. A high-volume disease according to CHAARTED criteria was diagnosed in CT and bone scans. There was no evidence of visceral metastasis. Additional immunohistochemical examinations of the resected tumor tissue showed positive expression of AE1/AE3, PSAP, PSMA and partially weakly positive for PSA, as well as nuclear positivity for AR (Fig. 6B). At that time, the cells were negative for synaptophysin and there was no evidence of a NE small-cell component. An intensified hormonal therapy with abiraterone was initiated. Subsequently, a serologic and morphologic tumor response was observed. After six months, however, local, PSA-negative progression of the primary tumor occurred (Fig. 6C). A biopsy showed extensive infiltrates of a NEPC with positivity for synaptophysin and negativity for PSA, AR, PSMA and NKX3.1 (Fig. 6D). The proliferation index Ki67 exceeded 95%. As shown in Fig. 6E-F, CTCs were detected at the time of metastatic PRAD diagnosis and showed strong expression of PRAD markers. Of note, *CHGA* and *CEACAM5* expression was also detected in the enriched cell fraction. This suggests that at least a subpopulation of cells acquired NE or amphicrine differentiation before treatment initiation. At the time point of PSA negative progression on first line therapy, the CTC count was still reduced compared to the pre-treatment sample. However, the number of detected NE markers increased to five while PRAD markers were reduced. Comparing both samples in the hierarchical clustering analysis, the progression was accompanied by a switch of the patient from the amphicrine to the NEPC cluster. This was consistent with the results of histological staining, which identified a PSA-negative tumor with expression of the NE marker synaptophysin. In conclusion, this case report highlights the suitability of our assay for the early detection of incipient NEPC and the added value compared to tissue biopsy alone.

## Discussion

AR-independent mPC is a deadly disease that is difficult to diagnose using existing biomarkers, but the emergence of NE features provides an opportunity to specifically detect emerging AR independence by Liquid biopsy analysis, which is a promising approach for the clinical management of patients [23]. Our results show that CTC enumeration is significantly higher in AVPC compared to mHSPC, and thus CTC detection may complement the criteria for AVPC detection by Aparicio [5]. Since CTC numbers are high but do not differ between AVPC and NEPC patients, molecular analysis of CTCs must be performed to extract relevant information. In support of previous studies [13, 14], we used gene expression analysis of bulk CTCs and NEPC samples were robustly predicted. We also found that *KLK3* gene expression is significantly decreased in CTCs isolated from patients with proven NEPC compared to AVPC patients, supporting that simple PSA measurements are not suitable for the detection and monitoring of therapy-induced NEPC.

The utility of EPCAM-based enrichment for CTCs from NEPC patients has also been discussed [23], and we compared EPCAM-dependent with size-based CTC enrichment to evaluate the impact of CTC enrichment method on gene expression analysis. While the detection of CTCs largely overlapped, NE markers were more frequently detected after AdnaTest-based CTC enrichment. Because EPCAM-based CTC enrichment is validated for the detection of small-cell lung cancer with comparable biology, we argue that EPCAM-based enrichment provides relevant information about the CTC subpopulation with NE traits, especially when combined with enrichment for EGFR and HER2 as in the AdnaTest [19, 21–24]. As reduced cell size is a NE feature [15, 25], we rather argue that relevant cells might be missed by size-dependent CTC enrichment.

Our panel consists of 22 AC and NE markers, allowing the identification of four major molecular clusters, including CTC^low^, AR^high^, NE, and amphicrine clusters that recapitulated the known phenotypes of treatment resistance [7]. In our study, most AVPC samples were characterized by high expression of AR and other AC markers, indicating that aberrant AR pathway activation might still be the main driver of tumor growth. The NEPC cluster was characterized by an upregulation of NE markers and a reduction of AC marker expression. Although a significant reduction in the expression of most AC markers was observed in the NEPC compared to the AVPC group, NEPC samples showed a dichotomous distribution of AC markers. This is in line with observations by Cancel et al. who found expression of AR and the androgen-regulated gene *NKX3-1* in more than half of NEPC samples, as those were often admixed with PRAD [26]. Expression of markers of terminal NE differentiation such as *CHGA*,* CEACAM5*, or *ACTL6B* was more often detected than upregulation of transcription factors driving NE differentiation such as *LMO3* or *NKX2-1*, suggesting that transcription factors might be less sensitive markers due to their comparably lower expression [7, 26, 27].

Conclusions about amphicrine disease with the combined expression of AC and NE markers are limited by the nature of the bulk CTC analysis on which our gene expression profiling is based. Since CTCs can be derived from different metastatic sites, amphicrine expression profiles may not necessarily be a consequence of an amphicrine tumor but could also be the superposition of expression profiles from separate luminal and NE metastases [28]. Interestingly similar subtypes were found in tissue samples by Labrecque et al. and their AR^low^ and double-negative clusters were most similar to the CTC^low^ cluster identified in the current analysis [7]. Taking CTC counts into account, this suggests that an AR^low^ subtype may be hidden in the CTC^low^ cluster, as specific positive markers for this subtype were not available. The analysis of methylation signatures in cell-free DNA offers the possibility to collect complementary data about the type of tumor. Recently, the targeted analysis of cfDNA methylation was successfully used to distinguish NEPC and other AR-independent subtypes from castration-resistant PRAD [29]. Specific methylation signatures and drivers of DNPC are also emerging and insights into the underlying biology will further advance the detection and monitoring of non-NE, AR-independent PC [30].

We trained a classifier to differentiate between pure NEPC and mHSPC or AVPC. While the number of NE markers already enabled subtype prediction, a random forest classifier allowed robust identification of NEPC samples with a sensitivity of 90.9% at 95.5% specificity. Previously, PCR-based detection of the NE markers *CHGA* and *SYP* in EPCAM-enriched CTCs was reported to have a specificity of 91% and a limited sensitivity of 51% for single sample classification. Notably, the integration of longitudinal samples from a single patient increased their per-patient predictive accuracy to 100% [14]. In contrast, our assay showed that the expression of a single marker, even at multiple time points, was not predictive of NEPC. Instead, patients with NEPC may have expressed different numbers of NE markers but were marker-positive at all time points because they were CTC positive. *SYP* was not included in our panel, as the background signal from leukocytes was too high [18, 19]. The inclusion of multiple NE markers improves single sample sensitivity and discrimination between pure NEPC and other subtypes of aggressive disease. The performance of the classifier heavily relies on the correct annotation of the training data. However, the intrinsic heterogeneity that is observed clinically and molecularly limits a definite categorization. Consequently, the classification of double-positive samples remains less accurate and prolonged follow-up of the training samples is required.

Our rapid and cost-effective PCR-based assay can be easily implemented into routine laboratory use, and automation of CTC enrichment offers the opportunity to further standardize the workflow. The lack of a validation cohort remains a limitation of our classifier and prospective sample collection is required to prove the value of our assay for the timely identification of therapy-induced NEPC in clinical practice. Although we have presented a case report highlighting the potential of our assay for early detection of therapy-induced NEPC, a systematic analysis of multiple patients is required to determine the lead time compared to clinical parameters.

## Conclusion

The present study demonstrates the value of transcriptional profiling of CTCs for monitoring metastatic prostate cancer patients. Our analysis revealed a high degree of inter-patient heterogeneity as well as distinct expression patterns of AR and NE markers, which allowed robust prediction of NEPC samples and identified treatment-relevant molecular subtypes. A longitudinal case study highlighted the advantages of our liquid biopsy-based analysis for the early detection of emerging therapy-induced NEPC. Thus, implementation of this test in clinical practice would help to identify the emergence of tNEPC and may allow early detection of resistance to androgen receptor pathway inhibitors and allocation to alternative treatments.

## Electronic supplementary material

Below is the link to the electronic supplementary material.

Supplementary Material 1

Supplementary Material 2

## Data Availability

No datasets were generated or analysed during the current study.

## Declarations

### Statement of translational relevance

Blood-based biomarkers of metastatic prostate cancer are urgently needed to identify patients with progression to aggressive and androgen-independent disease, and to monitor treatment response and emerging therapy resistance. We have developed a convenient PCR-based method to identify neuroendocrine prostate cancers based on their gene expression in circulating tumor cells. This test allows clinical monitoring of patients under androgen receptor-directed treatment and enables the early identification of progression to androgen-independent and therapy-induced neuroendocrine prostate cancer. In this way, suitable treatment strategies can be applied at an early stage before clinical symptoms and deterioration of general condition have occurred.
