# Supplementary material for: Transcriptional profiles of circulating tumor cells reflect heterogeneity and treatment resistance in advanced prostate cancer
Source: J Exp Clin Cancer Res. 2025 Apr 3;44:111. doi: 10.1186/s13046-025-03367-x (PMC11967125; doi:10.1186/s13046-025-03367-x)
Supplement: Supplementary file 2 — Supplementary Material 2 [file 13046_2025_3367_MOESM2_ESM.docx]

**Supplementary Information**

**Title:** Transcriptional Profiles of Circulating Tumor Cells Reflect Heterogeneity and Treatment Resistance in Advanced Prostate Cancer

**Authors:** Lina Bergmann^1,2^ , Sarah Greimeier^1,2^ , Sabine Riethdorf^1,2^ , Tina Rohlfing^2,3^ , Moritz Kaune^4^ Tobias Busenbender^2,3^ , Nadja Strewinski^2,3^ , Sergey Dyshlovoy^2,3^ , Simon Joosse^1,2,5^ , Sven Peine^6^, Klaus Pantel^1,2*,^ Gunhild von Amsberg^2,3,4*^, Stefan Werner^1,2,5*^

**Affiliations:**

^1^Institute of Tumor Biology, University Medical Center Hamburg-Eppendorf, Martinistrasse 52, 20246 Hamburg, Germany

^2^European Liquid Biopsy Society (ELBS), Hamburg, Germany

^3^Department of Hematology and Oncology, University Cancer Center Hamburg, University Medical Center Hamburg-Eppendorf, Germany

^4^Martini-Klinik, Prostate Cancer Center, University Medical Center Hamburg-Eppendorf, Germany

^5^Mildred Scheel Cancer Career Centre HaTriCS4, University Medical Centre Hamburg-Eppendorf, 20246 Hamburg, Germany

^6^Department of Transfusion Medicine, University Medical Center Hamburg-Eppendorf, Hamburg, Germany

* These authors contributed equally to this work.

**Running title**. CTC expression profiles in NEPC

**Supplementary Material and Methods**

**Human samples**. Blood samples were collected from PC patients between November 2020 and July 2023 at the University Medical Center Hamburg-Eppendorf. Patient characteristics are given in **Supplementary table S1**. Blood collection was conducted in accordance with the declaration of Helsinki and approved by the ethical commission of the city of Hamburg (Hamburger Ärztekammer, PV5392) and all patients provided informed consent. Patients were categorized by an experienced clinician based on clinical parameters and serum markers according to modified criteria defined by Epstein et al. and Aparicio et al. (1,2). Samples were pseudonymized in the clinic before transport to the research lab. As negative control, blood was collected in EDTA tubes from male donors at the blood donation facility at the Department for Transfusion medicine, University Medical Center Hamburg-Eppendorf. Blood samples from healthy donors were anonymized, but information about the age of the donors was available. All blood samples were processed within 3 h after blood collection.

**CTC enumeration**. The CellSearch system (Menarini Silicon Biosystems, Italy) was used to determine the number CTCs. Blood samples were fixed in CellSave tubes (Menarini Silicon Biosystems, Italy) and processed within 72 h. Briefly, EPCAM-positive cells were immunomagnetically enriched using the Cell Cellracks Autoprep System with the Circulating Tumor Cell Kit (both Menarini Silicon Biosystems, Italy) and stained for pan-cytokeratin and CD45. Stained, enriched cells were automatically scanned using the Celltracks Analyzer II and manually revised by an experienced user. Cells with an intact nucleus, positive cytokeratin staining and absent CD45 staining were counted as CTCs. When CTCs had a peri-nuclear dot-like cytokeratin staining, this was considered a small cell-like morphology.

**CTC enrichment for gene expression analysis**. Label-dependent CTC enrichment was done from 5 ml EDTA blood using the AdnaTest Prostate Cancer Select (Qiagen, Germany) according to the manufacturer’s instructions. In brief, whole blood was incubated with immunomagnetic beads directed against epithelial cell markers and the enriched cells were washed and lyzed. CTC lysates were stored at -80 °C until further processing.

For size-dependent CTC enrichment, the Parsortix PR1 device (Angle plc, UK) was used. According to the manufacturer’s instructions, the Parsortix device was primed with the *PX2_PF* program and a GEN 6.5 cassette was inserted. The CTCs were enriched using the S50F or the S99F protocol and harvested using the *PX2_H* program with the *Further Flush* option. Cells were either harvested directly into a cytospin funnel or into a reaction tube. The cells were spun down and resuspended in AdnaTest lysis buffer. Lysates and cytospin slides were stored at -80 °C.

**Cell lines**. The following cell lines representing different stages of PCa progression were purchased from ATCC: LNCaP (RRID: CVCL0395), VCaP (RRID: CVCL22359), NCI-H660 (RRID: CVCL1576), DU145 (RRID: CVCL0105), PC3 (RRID: CVCL0035), LASCPC-01 (RRID: CVCLUE17), NCI-H209 (RRID: CVCL1525). Their expression profiles with regard to adenocarcinoma and NE markers is shown in **Fig. 2A**. All cell lines were established from male patients cultivated in their suggested cell culture media for 20-30 passages. Cell lines were authenticated by tandem repeat profiling and were monthly tested for mycoplasma contamination using the Venor®GeM detection kit (Minerva Biolabs, Germany). For the validation of the CTC marker panel, a single cell solution was prepared and single cells were picked under a phase-contrast microscope and 25 individual cells were transferred to 7.5 ml healthy donor blood or AdnaTest lysis buffer. These positive controls were analyzed like the patient samples.

**Gene expression analysis**. Transcripts for gene expression analysis were selected after literature search on PubMed (National Center for Biotechnology Information) using the search terms “neuroendocrine prostate cancer” and “small cell prostate cancer”. Because enriched CTCs are contaminated with leukocytes, the expression of these genes was examined in RNA immune cell data sets in *The Human Protein Atlas* (v21.0.proteinatlas.org)(3).

RNA was isolated from cell lines using the NucleoSpin RNA Mini kit (Macherey & Nagel, Germany) according to the manual and cDNA was generated using the RevertAid First Strand cDNA Synthesis Kit (Thermo Scientific, United States). Gene expression was measured by real-time quantitative PCR using the C1000 Touch CFX96 thermocycler (Bio-Rad Laboratories, United States). Single reactions were performed with TATAA Probe or SYBR GrandMaster Mix (TATAA Biocenter, Sweden) and a final concentration of 0.4 µM per primer (see **Supplementary Table S2** for list of all primers). The PCR protocol included 40 cycles with an annealing temperature of 60 °C.

Gene expression analysis of AdnaTest lysates was conducted as previously published (4). mRNA purification and cDNA synthesis were performed using the AdnaTest Prostate Cancer Detect and the Sensiscript RT Kit (both Qiagen, Germany) according to the manufacturer’s instructions. Transcripts of interest were preamplified with a mix of 9 to 15 single assays for transcripts of interest and housekeeping genes at a final concentration of 50 nM per assay. Preamplification reactions were diluted 1:8 and subjected to individual qPCR analysis per assay. A list of all primer and probe pairs is given in **Supplementary table S2**. The ValidPrime assay (TATAA Biocenter, Sweden) was included to correct for the presence of genomic DNA. The TATAA Interplate Calibrator was used to calibrate C_q_ values from individual plates according to the manufacturer’s instructions. For data evaluation, raw C_q_ values were first calibrated using the interplate calibrator and, when necessary, corrected for the presence of genomic DNA using the ValidPrime assay C_q_ value. According to the AdnaTest manual a cut-off of 35 was chosen and all corrected Cq values ≥ 35 were defined negative. Corrected C_q_ values of each sample were normalized using the mean C_q_ value of *ACTB* and *GAPDH*. Based on the results of blood samples from ten healthy individuals, a threshold was defined as the mean ΔC_q_ value minus one standard deviation to correct for potential signals from contaminating leukocytes. Normalized and healthy donor corrected Cq values were reversed by subtracting from 26 (rounding up the highest ΔC_q_) and transcripts without a signal were set to 0.

***In silico* validation**. Published RNA sequencing data sets for PCa tissue samples were downloaded from cBioPortal (cbioportal.org)(5-7). For the Neuroendocrine Prostate Cancer data set (Multi-Institute, Nat Med 2016), gene expression values were downloaded as log(FPKM) values(8). For the Metastatic Prostate Adenocarcinoma data set (SU2C/PCF Dream Team, PNAS 2019), gene expression values were downloaded as z-scores relative to all samples (9). Hierarchical clustering on published data sets was performed with the *complete* method and *spearman* as distance measure.

**Classification of NEPC samples based on CTC gene expression**. Only CTC-positive samples from individual patients were included in the supervised analysis. The R packages ranger (V 0.16.0) and caret (V 6.0-94) were used for the random forest analysis. mtry was set to 2, importance was set to *impurity* and splitrule was set to *extratrees*, while all other parameters were left at their default values. Leave-one-out cross-validation was included to calculate the out-of-bag error rate.

**Statistical analysis**. CTC counts and differential gene expression between the groups were analyzed using Kruskal-Wallis test and the Wilcoxon rank sum test together with Dunn’s correction for multiple testing for pair-wise comparison. Chi-squared test and Fisher’s exact test were used to assess differences between categorical variables, such as the positivity of a certain marker per group, while clustering of based on gene expression profiles was performed using the *ward.D* method and *euclidean* as distance measure.

**Data availability**. The data generated in this study are available upon request from the authors. Tissue expression data analyzed in this study were obtained from cBioPortal https://www.cbioportal.org/study/summary?id=nepc_wcm_2016 and https://www.cbioportal.org/study/summary?id=prad_su2c_2019.

**Supplementary Table S2: Oligonucleotide Primers.** Sequences of all custom primer pairs and probes; for commercial assays catalogue numbers are provided, since the sequences are not published.

| Transcript | Oligo type | Sequence |
| --- | --- | --- |
| ACTL6B (10) | Forward Primer | GGC CTG TTT GAT CCC TCG AA |
|  | Reverse Primer | GGT GAC AAT GAC ACT CCC GT |
| ASCL1 | Forward Primer | TCG CCG GTC TCA TCC TAC TC |
|  | Reverse Primer | GTT GTG CGA TCA CCC TGC TT |
|  | Probe | AGG AGC TTC TCG ACT TCA CCA ACT GG - FAM/ZEN |
| CEACAM5 | Forward Primer | GGG ACC TAT GCC TGT TTT GTC |
|  | Reverse Primer | AGA GAC CAG GAG AAG TTC CAG A |
|  | Probe | TTC CAT AGT CAA GAG CAT CAC AGT CTC TGC - FAM/ZEN |
| CHGA | Forward Primer | TCC CTG TGA ACA GCC CTA TG |
|  | Reverse Primer | AAG GAT CCG TTC ATC TCC TCG G |
|  | Probe | CTC CGA CAC ACT TTC CAA GCC CAG CCC - FAM/ZEN |
| FOXA2 | Forward Primer | CGG TGA AGA TGG AAG GGC A |
|  | Reverse Primer | CAT GTT GCT CAC GGA GGA GT |
|  | Probe | AGC CGT CCG ACT GGA GCA GC - FAM/ZEN |
| KRT6A | Forward Primer | TAG TGC CCT CAC TTC TTC TCT CTC |
|  | Reverse Primer | GCT CAG CCT CAG AGA TAG AAC AC |
|  | Probe | TGT AAT CAC CAC TGG AGC TTC ACT GTT - FAM/ZEN |
| LIN28B (11) | Forward Primer | TGT AGT CTA CCT CCT CAG CCA A |
|  | Reverse Primer | ATT CTG CTT CCT GTC TTC CCT G |
| LMO3 (12) | Forward Primer | TCT GAG GCT CTT TGG TGT AAC G |
|  | Reverse Primer | CCA GGT GGT AAA CAT TGT CCT TG |
| NKX2-1 | Forward Primer | CGT ACC AGG ACA CCA TGA G |
|  | Reverse Primer | ATG CCG CTC ATG TTC ATG C |
|  | Probe | CCA TCT CCC GCT TCA TGG GC - FAM/ZEN |
| NKX3-1 | Forward Primer | CCC ACA CTC AGG TGA TCG AG |
|  | Reverse Primer | GAG CTG CTT TCG CTT AGT CTT |
| PCSK1 | Forward Primer | CCG ACC AGA GAA TCA CGA GC |
|  | Reverse Primer | ACC AGG TGC TGC ATA TCT CG |
|  | Probe | CCA GAG CGA AGA TGC CAG CAG CC - FAM/ZEN |
| PEG10 | Forward Primer | GCC TCC ATC CCC ACA GAA GTG AAG C |
|  | Reverse Primer | CAC TCT TAT GGC CGG TGT GCT TGG A |
|  | Probe | CCC AAC CGT CAC CCT GGG TCC CGA CTG CCC - FAM/ZEN |
| POU3F2 | Forward Primer | GTA ACT GTC AAA TGC GCG GC |
|  | Reverse Primer | GAG GTG AGC AGG CTG TAG TG |
|  | Probe | CGG TCG CCA TGA CTC TCG GAG CC - FAM/ZEN |
| SOX11 (13) | Forward Primer | CCA GGA CAG AAC CAC CTG AT |
|  | Reverse Primer | CCC CAC AAA CCA CTC AGA CT |
| SRRM4 | Forward Primer | GCC CAT CGC CTG TCA AGA AA |
|  | Reverse Primer | TTT GGG CTA GAG GAG CTG TG |
|  | Probe | AAA GTT CCA AGA AAC ACA AGC GAC G - FAM/ZEN |
| Commercial Assays | | |
| Transcript | Catalogue number | Company |
| ACTB | qA-01-0104P | TATAA Biocenter |
| AR | qA-01-0364P | TATAA Biocenter |
| AR-V7 | qA-01-0368P | TATAA Biocenter |
| CCND1 | qA-01-0203P | TATAA Biocenter |
| CD45/PTPRC | qA-01-0240P | TATAA Biocenter |
| EGFR | qA-01-0211P | TATAA Biocenter |
| EPCAM | qA-01-0212P | TATAA Biocenter |
| FOLH1 | qA-01-0357P | TATAA Biocenter |
| GAPDH | qA-01-0101P | TATAA Biocenter |
| HOXB13 | qA-01-0889P | TATAA Biocenter |
| KLK3 | qA-01-0356P | TATAA Biocenter |
| KRT19 | qA-01-0225P | TATAA Biocenter |
| PROM1 | qA-01-0371P | TATAA Biocenter |
| RAI2 | qA-01-0890P | TATAA Biocenter |
| TACSTD2 | qA-01-0372P | TATAA Biocenter |

**Supplementary Figure S1**


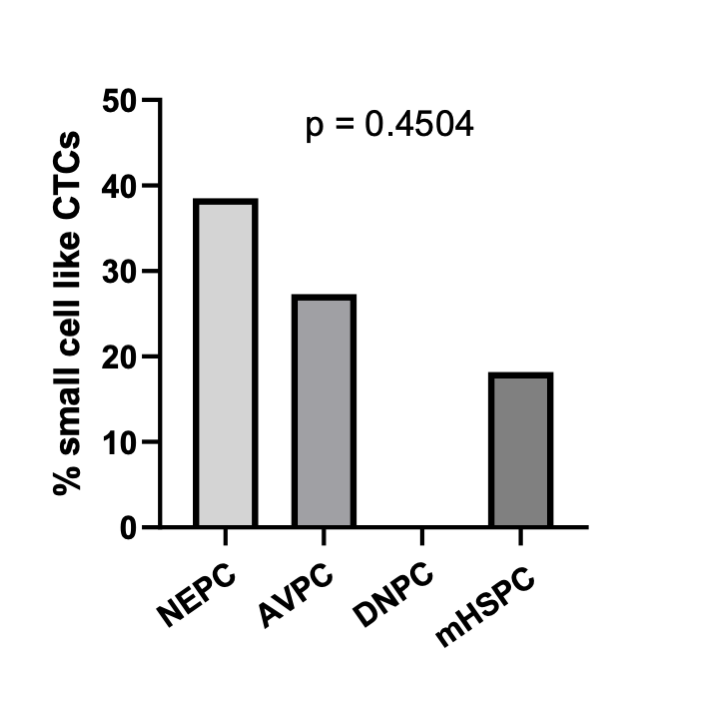


**Supplementary Figure S1: Frequency of small cell like CTCs in CellSearch**. The frequency of samples with CTCs that have a small cell morphology was compared between NEPC, AVPC, DNPC and mHSPC patients; Chi-squared test was used for significance testing.

**Supplementary Figure S2**


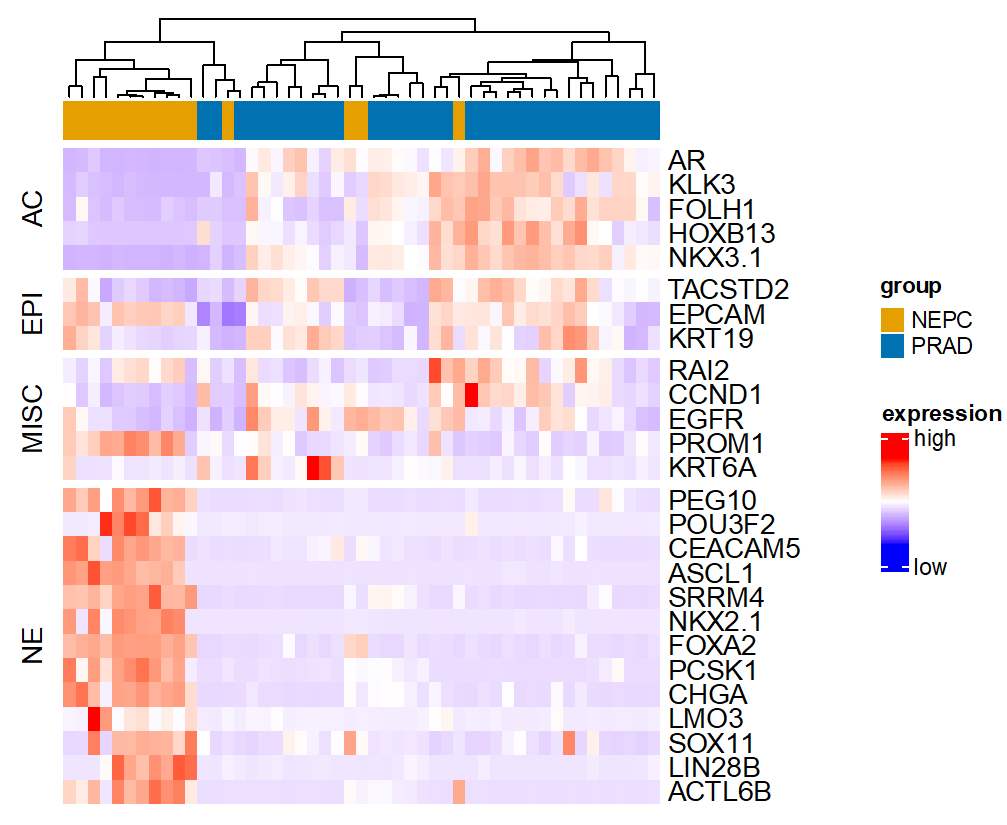


**Supplementary Figure S2: Hierarchical clustering of published CRPC tissue samples based on marker panel**. RNA expression data from the Neuroendocrine Prostate Cancer Data Set (Multi-Institute, Nat Med 2016 (8)) were downloaded from cBioPortal and the samples were clustered based on the relative expression of the marker panel, the top annotations shows the clinical group of the samples based on the annotation in the original data set.

**Supplementary Figure S3**


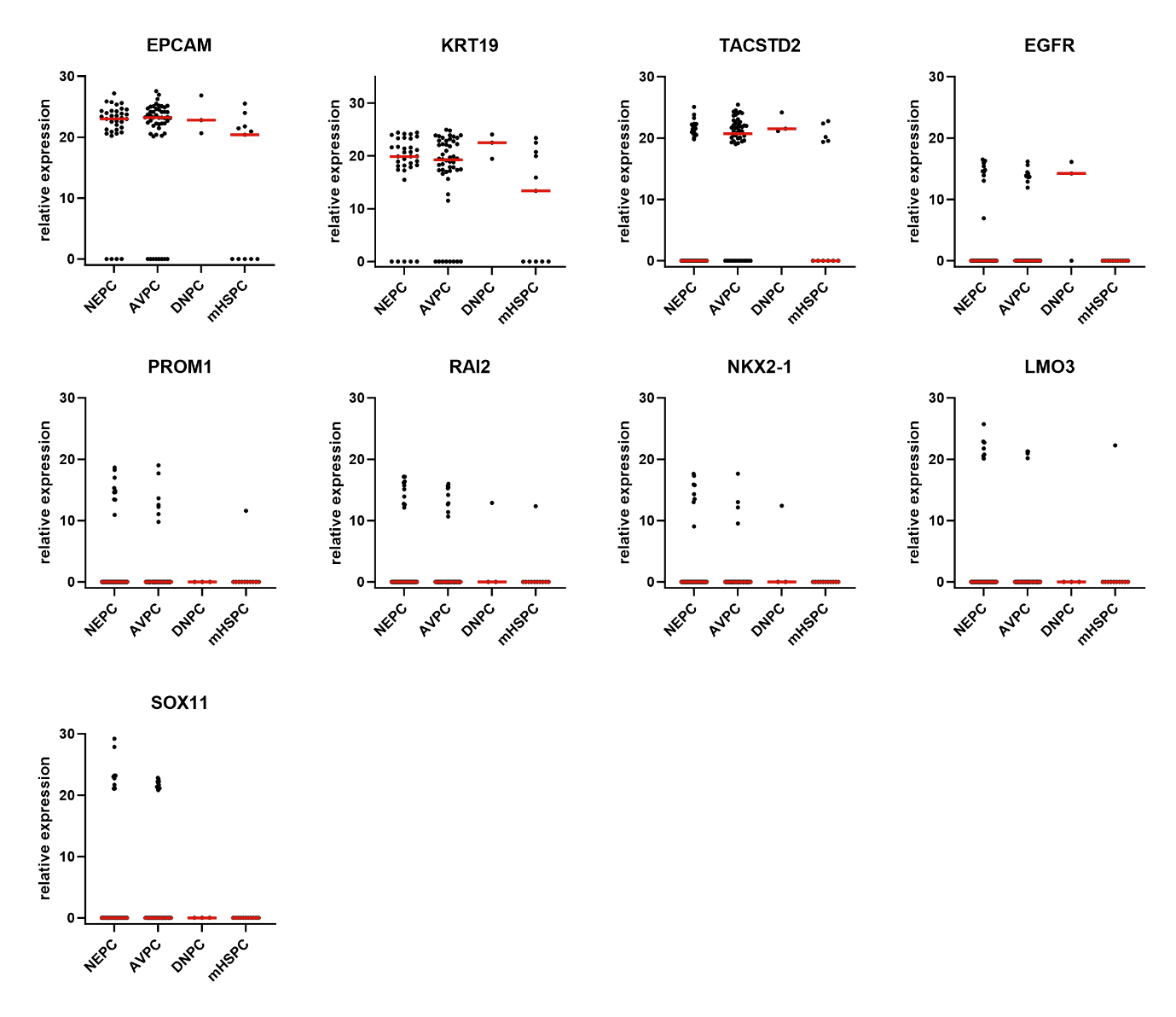


**Supplementary Figure S3: Expression of indicated genes in enriched CTC fractions from advanced PC patients**. CTCs were enriched from blood samples of PC patients using the AdnaTest Prostate Cancer Select and a panel of selected markers was detected following multiplex pre-amplification of isolated RNA; samples were grouped according to clinical and histopathological parameters into neuroendocrine PC (NEPC), aggressive variant PC (AVPC), double-negative PC (DNPC) and hormone-sensitive PC (mHSPC); normalized gene expression is shown as reversed ΔCq and the Kruskal Wallis Test together with Wilcoxon rank sum test and Dunn’s correction for multiple testing for pair-wise comparison.

**Supplementary Figure S4**

**Supplementary Figure S4: Correlation of the gene expression of single markers.** Normalized gene expression of all marker transcripts irrespective of the patient group was subjected to correlation analysis by Spearman correlation coefficient, significant correlations are shown as dots with the color resembling the correlation coefficient: blue – positive correlation, red – negative correlation.

**Supplementary Figure S5**

**
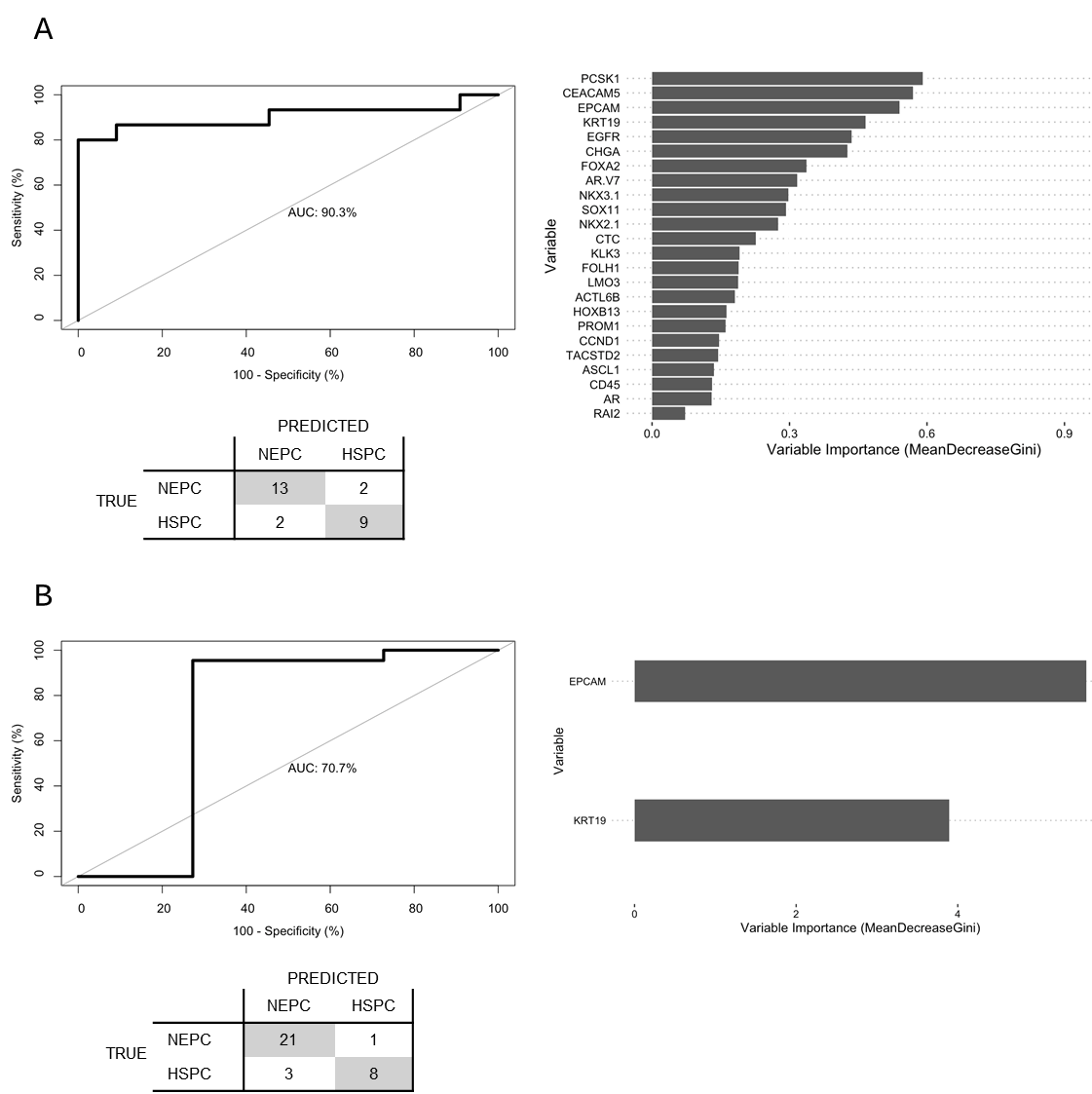
**

**Supplementary Figure S5:** **Classification of NEPC samples based on selected gene expression features in CTCs considering CTC counts.** A: Random forest classification of NEPC vs HSPC samples, including CTC count as an additional feature (compared to Fig. 5 only 26 samples are included, as CellSearch results were not available for all samples); ROC analysis of the random forest classifier trained on normalized gene expression data of NEPC and mHSPC samples from individual patients positive for CTCs; results of the leave-one-out cross-validation included in the classifier training; variable importance of the individual markers included in the classifier; B: Random forest analysis comparable to Fig. 5, but considering only EPCAM and KRT19 CTC expression as features.

**References**

1. Epstein JI, Amin MB, Beltran H, Lotan TL, Mosquera JM, Reuter VE*, et al.* Proposed morphologic classification of prostate cancer with neuroendocrine differentiation. Am J Surg Pathol **2014**;38:756-67

2. Aparicio AM, Harzstark AL, Corn PG, Wen S, Araujo JC, Tu SM*, et al.* Platinum-based chemotherapy for variant castrate-resistant prostate cancer. Clin Cancer Res **2013**;19:3621-30

3. Uhlen M, Karlsson MJ, Zhong W, Tebani A, Pou C, Mikes J*, et al.* A genome-wide transcriptomic analysis of protein-coding genes in human blood cells. Science **2019**;366

4. Besler K, Węglarz A, Keller L, von Amsberg G, Bednarz-Knoll N, Offermann A*, et al.* Expression Patterns and Corepressor Function of Retinoic Acid-induced 2 in Prostate Cancer. Clin Chem **2022**;68:973-83

5. Cerami E, Gao J, Dogrusoz U, Gross BE, Sumer SO, Aksoy BA*, et al.* The cBio cancer genomics portal: an open platform for exploring multidimensional cancer genomics data. Cancer Discov **2012**;2:401-4

6. Gao J, Aksoy BA, Dogrusoz U, Dresdner G, Gross B, Sumer SO*, et al.* Integrative analysis of complex cancer genomics and clinical profiles using the cBioPortal. Sci Signal **2013**;6:pl1

7. de Bruijn I, Kundra R, Mastrogiacomo B, Tran TN, Sikina L, Mazor T*, et al.* Analysis and Visualization of Longitudinal Genomic and Clinical Data from the AACR Project GENIE Biopharma Collaborative in cBioPortal. Cancer Res **2023**;83:3861-7

8. Beltran H, Prandi D, Mosquera JM, Benelli M, Puca L, Cyrta J*, et al.* Divergent clonal evolution of castration-resistant neuroendocrine prostate cancer. Nat Med **2016**;22:298-305

9. Abida W, Cyrta J, Heller G, Prandi D, Armenia J, Coleman I*, et al.* Genomic correlates of clinical outcome in advanced prostate cancer. Proc Natl Acad Sci U S A **2019**;116:11428-36

10. Ahn LY, Coatti GC, Liu J, Gumus E, Schaffer AE, Miranda HC. An epilepsy-associated ACTL6B variant captures neuronal hyperexcitability in a human induced pluripotent stem cell model. J Neurosci Res **2021**;99:110-23

11. Lovnicki J, Gan Y, Feng T, Li Y, Xie N, Ho CH*, et al.* LIN28B promotes the development of neuroendocrine prostate cancer. J Clin Invest **2020**;130:5338-48

12. Ling Z, Long X, Wu Y, Li J, Feng M. LMO3 promotes proliferation and metastasis of papillary thyroid carcinoma cells by regulating LIMK1-mediated cofilin and the β-catenin pathway. Open Med (Wars) **2022**;17:453-62

13. Huang J, Ji EH, Zhao X, Cui L, Misuno K, Guo M*, et al.* Sox11 promotes head and neck cancer progression via the regulation of SDCCAG8. J Exp Clin Cancer Res **2019**;38:138
